# Pain and health-related quality of life in adolescents and the mediating role of self-esteem and self-efficacy: a cross-sectional study including adolescents and parents

**DOI:** 10.1186/s40359-021-00629-z

**Published:** 2021-08-30

**Authors:** Hilde Timenes Mikkelsen, Kristin Haraldstad, Sølvi Helseth, Siv Skarstein, Milada Cvancarova Småstuen, Gudrun Rohde

**Affiliations:** 1 Department of Health and Nursing, Faculty of Health and Sport Sciences, University of Agder, PO Box 422, 4604 Kristiansand, Norway; 2 Department of Nursing and Health Promotion, Faculty of Health Sciences, Metropolitan University, Oslo, Norway; 3 Department of Clinical Research, Sorlandet Hospital, Kristiansand, Norway

**Keywords:** Persistent pain, Health-related quality of life, Adolescents, Parents, Self-efficacy, Self-esteem, Resilience, Mediation

## Abstract

**Background:**

To promote health-related quality of life (HRQOL) in adolescents with pain, it is important to study factors associated with pain. This study aimed to describe selected factors and pain in 14–15-year-old adolescents and their parents, to assess how these factors are associated with adolescent pain groups, and to explore whether the relationship between pain intensity and HRQOL in adolescents with persistent pain is mediated by self-esteem and self-efficacy.

**Methods:**

A cross-sectional study was performed among 508 dyads of adolescents (14–15 years) and parents in a school-based setting. Among these, 148 adolescents had persistent pain. We explored the following variables: HRQOL, pain, self-efficacy, self-esteem, sleep, loneliness, stress and sociodemographic variables. All variables were assessed with well-validated instruments. HRQOL was measured with KIDSCREEN-27. Analyses included Chi-square, ANOVA, Mann–Whitney U tests, Kruskal–Wallis and the PROCESS macro method for mediation analyses.

**Results:**

Adolescents with pain reported significantly higher levels of stress, loneliness and lack of sleep and lower levels of self-efficacy, self-esteem and HRQOL compared to adolescents without pain. More girls than boys reported pain. Adolescents with persistent pain scored significantly worse on self-esteem, stress, loneliness, lack of sleep, school absence, pain and HRQOL compared to adolescents with shorter pain duration. Adolescent pain groups did not differ significantly considering parental factors. However, more adolescents with persistent pain reported that someone in their family had pain. The associations between pain intensity and the HRQOL subscales in adolescents with persistent pain were completely mediated by self-esteem, but not by self-efficacy. The highest degree of mediation was estimated for the HRQOL subscale school environment (indirect effect = 73.5%).

**Conclusions:**

Our findings highlight the complexity within adolescent pain, demonstrating that adolescents with pain differ from adolescents without pain when it comes to gender, school absence, factors within-person and between-persons. Longer pain duration makes adolescents more vulnerable. We confirm the importance of resilience factors for HRQOL but indicate that self-esteem is more important than self-efficacy. To promote HRQOL in adolescents with persistent pain, a strengthening of both their self-esteem and self-efficacy is recommended. We highlight the need for an individual, holistic approach to adolescent pain.

**Supplementary Information:**

The online version contains supplementary material available at 10.1186/s40359-021-00629-z.

## Introduction

Pain problems in adolescents have increased during the last 2 decades and is recognized as a substantial public health challenge in industrialized countries [1, 2]. Negative consequences of adolescent pain include peer relationship problems, sleeping problems, avoidance of activities and sports, school absenteeism, an increased risk of recurrent pain in adulthood and a decreased health-related quality of life (HRQOL) [3–9]. The term HRQOL is a multidimensional construct that includes the individual’s subjective perspectives on the physical, psychological, social and functional aspects of health [10]. By measuring HRQOL in adolescents with pain, one can gain insight into adolescents’ subjective experiences of pain and how pain affects different dimensions of their lives [11].

According to the International Association for the Study of Pain, pain is always a personal experience that is influenced by biological, psychological, and social factors [12]. Adolescents communicate pain more and less clearly, and causal factors range from identifiable to very diffuse [10]. Sociodemographic factors [e.g. age, gender, ethnicity, socioeconomic status (SES)] and factors within-person (e.g. stress, self-efficacy, self-esteem, sleeping problems) and between-persons (e.g. parents/family, loneliness, the school situation) contribute to and affect adolescent pain from both a resilience perspective and a risk perspective [2, 4, 8, 13–22]. Further, studies have shown that a history of pain in their parents may increase adolescent risk for chronic pain [14, 15, 23].

Internationally comparable data suggest persistent or chronic pain among adolescents in different countries is highly prevalent [24]. Chronic pain is defined as persistent or recurrent pain lasting longer than 3 months [25]. Persistent pain has consequences for the individual, their family and the society at large [19, 26–28]. More knowledge on factors that characterize adolescents with and without pain in a non-clinical population is necessary to better understand the cause of pain problems and find the best strategies to help adolescents with pain. Moreover, knowledge of whether there are factors in adolescents with persistent pain that differ from factors in adolescents with pain lasting less than 3 months is needed.

Adolescent pain research has mainly focused on maladjustment and risk factors. Thus, shifting the focus to protective and resilience factors has been recommended [18, 29, 30]. The term *resilience* refers to the state of an individual having a relatively good psychological outcome despite the presence of risk factors [31]. Both self-esteem and self-efficacy are considered resilient factors [22, 32, 33]. Self-esteem represents one’s positive or negative attitude toward oneself [34], whereas self-efficacy represents a self-confident view of one’s capability to deal with certain stressors in life [32]. Studies have shown that self-efficacy and self-esteem have a positive impact on both pain and HRQOL in adolescents [18, 35–43]. Higher levels of self-efficacy and self-esteem may protect an adolescent from experiencing pain, it can help to positively adapt and cope with pain, and it is associated with higher levels of HRQOL despite having pain [18, 22, 38, 41, 42].

There is a wide variety and complexity within adolescent pain, and it is therefore important to understand pain in light of a holistic model [11], such as the multidimensional biobehavioral model of pediatric pain [44, 45]. This model illustrates that pain may arise from several precipitants (such as disease, injury or stress) and that potentially modifiable intervening variables can influence both pain and HRQOL. According to this model, these intervening variables are biological predispositions (e.g. genetics, age, gender), family environment (e.g. family functioning, family pain models), perceived social support, cognitive appraisal and coping strategies [44, 45]. Inspired by the model, the present study focused on selected factors in adolescents (sociodemographic characteristics, self-efficacy, self-esteem, loneliness, stress, sleep, HRQOL and pain characteristics) and in their parents (sociodemographic characteristics, HRQOL, parental pain characteristics) that previous research has identified as being associated with adolescent pain [2, 4, 8, 13–22]. These factors can be viewed as both precipitants and intervening variables within the model. More knowledge of these factors in adolescents might shed light on the complexity of adolescent pain. Moreover, because we consider self-efficacy and self-esteem as highly relevant intervening factors within the model, we focused on the possible mediating effect of self-efficacy and self-esteem on the relationship between pain and HRQOL in adolescents with persistent pain.

This study aimed to describe selected sociodemographic- and psychosocial factors and pain in 14–15-year-old adolescents and their parents. Further, to assess how these factors were associated with adolescent pain groups (no pain, pain lasting less than 3 months, persistent pain). Lastly, to explore whether the relationship between pain intensity and HRQOL in adolescents with persistent pain is mediated by self-esteem and/or self-efficacy.

## Methods

### Sample and data collection

The present study was conducted from November 2018 to April 2019 in the south-eastern part of Norway as part of the “Start Young—Quality of Life and Pain in Generations” study [43]. Schools covering ninth grade in elementary school (students aged 14–15 years) were stratified according to geographical region, school size, urban and rural districts. Two schools were randomly selected from each such stratum and invited to participate. Schools that declined were replaced by other schools selected according to the same criteria. In total, 22 schools that varied in size and localization participated. Project members visited participating schools to provide the adolescents with verbal and written information about the study. Parents received written information. Active informed consent was obtained from both adolescents and their parents. Potential participants in the study were 1663 adolescent–parent dyads from the participating schools. In total, 696 adolescents (41.8%) and 561 parents (33.7%) filled in the questionnaire. In this study, we included the 508 adolescent–parent dyads (30.5% of the invited) with responses from both adolescents and one of their parents. The response rate varied across schools, from 2.9 to 71.1%.

The data collection was done through a web-based questionnaire. Adolescents completed the questionnaire in classroom during school hours. Parents received a mail with a safe link to the questionnaire and completed the questionnaire at home. A safe data server was used to store the collected data [46]. Information from the parents’ consent form enabled us to link the questionnaires from the adolescents with their parents’ questionnaires by creating a mutual ID number.

All study procedures were approved by the Norwegian Centre for Research Data (Ref:60981).

### Measures

The study used an electronic survey tool that consecutively administered the following questionnaires. Most questions included a neutral option. This resulted in all items being answered. Thirteen variables that had several categories were recoded into fewer categories. This is explained in Tables 1 and 2. All questionnaires that use sum scales showed satisfactory values of Cronbach’s alpha above 0.7 in the present study (Additional file 1).

**Table 1:** Sociodemographic characteristics in adolescents and their parents by adolescent pain group (N = 508)

	Total (N = 508)	No pain (n = 124)	Pain < 3 months (n = 236)	Persistent pain (n = 148)	*p* value
*Adolescent characteristics*					
Gender, N (%)					**< .001**^**1,2**^
Female	281 (55.3)	42 (33.9)	141 (59.7)	98 (66.2)	
Male	227 (44.7)	82 (66.1)	95 (40.3)	50 (33.8)	
Age, median (min, max)	14.0 (14.0, 15.0)	14.0 (14.0, 15.0)	14.0 (14.0, 15.0)	1.0 (14.0, 15.0)	.447
Adult members of the household, N (%)^a^					.204
Both parents	381 (75.0)	99 (79.8)	176 (74.6)	106 (71.6)	
Alternates between two parents	68 (13.4)	15 (12.1)	27 (11.4)	26 (17.6)	
One parent and/or other caregivers	59 (11.6)	10 (8.1)	33 (14.0)	16 (10.8)	
Parents’ work status, N (%)^b^					.246
Both parents are working	414 (81.5)	104 (83.9)	196 (83.1)	114 (77.0)	
One parent is working	94 (18.5)	20 (16.1)	40 (16.9)	34 (23.0)	
School absence for the previous 3 months, N (%)^c^					**.016**^**2**^
No absence	180 (35.4)	54 (43.5)	78 (33.1)	48 (32.4)	
1–4 days	259 (51.0)	60 (48.4)	129 (54.7)	70 (47.3)	
≥ 5 days	69 (13.6)	10 (8.1)	29 (12.3)	30 (20.3)	
*Parent characteristics*					
Gender, N (%)					.568
Female	393 (77.4)	95 (76.6)	179 (75.8)	119 (80.4)	
Male	115 (22.6)	29 (23.4)	57 (24.2)	29 (19.6)	
Age, mean (SD)	45.2 (4.9)	45.4 (4.5)	45.2 (4.9)	45.2 (5.1)	.923
Marital status, N (%)^d^					.195
Married/cohabitant	422 (83.1)	109 (87.9)	195 (82.6)	118 (79.9)	
Single/divorced	86 (16.9)	15 (12.1)	41 (17.4)	30 (20.3)	
Education level, N (%)^e^					.697
≤ 12 years and/or certificate of apprenticeship	127 (25.0)	28 (22.6)	59 (25.0)	40 (27.0)	
13–15 years (< 4 years of higher education)	129 (25.4)	32 (25.8)	65 (27.5)	32 (21.6)	
≥ 16 years (≥ 4 years of higher education)	252 (49.6)	64 (51.6)	112 (47.5)	76 (51.4)	
Work status, N (%)					.297
Yes, full time	375 (73.8)	83 (66.9)	181 (76.7)	111 (75.0)	
Yes, part time	91 (17.9)	30 (24.2)	36 (15.3)	25 (16.9)	
No, not employed	42 (8.3)	11 (8.9)	19 (8.1)	12 (8.1)	
Household income, N (%)^f^					.527
≤ 450,000 NOK/year	44 (8.7)	9 (7.3)	20 (8.5)	15 (10.1)	
451,000–750,000 NOK/year	88 (17.3)	18 (14.5)	49 (20.8)	21 (14.2)	
751,000–1,000,000 NOK/year	116 (22.8)	33 (26.6)	51 (21.6)	32 (21.6)	
> 1,000,000 NOK/year	260 (51.2)	64 (51.6)	116 (49.2)	80 (54.1)	

Bold values indicates statistically significant differences between the groups (*p* ≤ 0.05)

Continuous variables analyzed with ANOVA with Tukey’s HSD post hoc test or Kruskal–Wallis and Mann–Whitney U tests between pairs of groups

Categorical variables analyzed with x^2^-test. Significant differences between the marked groups: ^1^No pain versus Pain < 3 months, ^2^No pain versus Persistent pain and ^3^Pain < 3 months versus Persistent pain. *p* values marked with bold indicate statistically significant differences between the groups (*p* ≤ 0.05)

The pain group variable was recoded into three categories: “No pain,” “Pain < 3 months” (only once, < 1 month, 1–3 months) or “Persistent pain” (> 3 months, > 6 months, > 12 months)

*SD* standard deviation, *PCS* physical component summary, *MCS* mental component summary

^a^The variable was recoded into three categories: “Both parents,” “Alternates between two parents” or “One parent and/or other caregivers” (one parent and one step-parent, one parent, other caregivers)

^b^The variable was dichotomized as “Both parents are working” or “One parent is working” (one parent is working, no parents are working)

^c^The variable was recoded into three categories: “No absence,” “1–4 days” or “≥ 5 days” (5–7 days, 8–10 days, > 10 days)

^d^The variable was dichotomized as “Married/cohabitant” or “Single/divorced” (single, divorced, widowed)

^e^The variable was recoded into three categories: “≤ 12 years and/or certificate of apprenticeship” (9 years, 10–11 years, 12 years, certificate of apprenticeship), “13–15 years (< 4 years of higher education)” or “≥ 16 years (≥ 4 years of higher education)”

^f^the variable was recoded into four categories: “≤ 450,000 NOK/year” (< 250,000 NOK/year, 250,000–450,000 NOK/year), “451,000–750,000 NOK/year,” “751,000–1,000,000 NOK/year” or “> 1,000,000 NOK/year.”

**Table 2:** Descriptive data for adolescent- and parent pain-related factors by adolescent pain group (N = 508)

	Total (N = 508)	No pain (n = 124)	Pain < 3 months (n = 236)	Persistent pain (n = 148)	*p* value
*Adolescent characteristics*					
Physical well-being, mean (SD)^a,b^	47.4 (9.3)	51.2 (10.0)	47.4 (8.4)	44.2 (9.4)	**< .001**^**1,2,3**^
Psychological well-being, mean (SD)^a,b^	46.6 (8.6)	52.3 (8.2)	46.1 (7.0)	42.7 (8.6)	**< .001**^**1,2,3**^
Autonomy and parent relations, mean (SD)^a,b^	52.8 (8.7)	56.7 (9.0)	52.4 (8.0)	50.2 (8.6)	**< .001**^**1,2,3**^
Social support and peers, mean (SD)^a,b^	48.3 (8.4)	50.6 (8.3)	48.1 (8.2)	46.7 (8.6)	**0.004**^**1,2**^
School environment, mean (SD)^a,b^	48.2 (8.8)	52.5 (9.6)	47.6 (8.2)	45.6 (7.9)	**< .001**^**1,2**^
Self-efficacy, mean (SD)^c^	3.1 (0.4)	3.2 (0.4)	3.1 (0.4)	3.0 (0.4)	**< .001**^**1,2**^
Self-esteem, median (min, max)^d^	3.0 (1.0, 4.0)	3.5 (1.7, 4.0)	3.0 (1.0, 4.0)	3.0 (1.0, 4.0)	**< .001**^**1,2,3**^
Loneliness, median (min, max)^e^	12 (8, 32)	11 (8, 21)	13 (8, 32)	14 (8, 32)	**< .001**^**1,2,3**^
Stress, mean (SD)^f^	0.29 (0.16)	0.20 (0.12)	0.29 (0.15)	0.36 (0.17)	**< .001**^**1,2,3**^
Frequency of enough sleep N (%)^g^					**< .001**^**1,2,3**^
Usually/always	329 (64.9)	101 (82.1)	152 (64.4)	76 (51.4)	
Sometimes/rarely	178 (35.1)	22 (17.9)	84 (35.6)	72 (48.6)	
Problems with sleepiness N (%)^h^					**< .001**^**1,2,3**^
No	213 (42.0)	81 (65.9)	98 (41.5)	34 (23.0)	
Yes	294 (58.0)	42 (34.1)	138 (58.5)	114 (77.0)	
*Parent characteristics*					
RAND-36 PCS, mean (SD)^i^	51.5 (9.0)	52.0 (8.6)	51.4 (9.1)	51.4 (9.1)	.803
RAND-36 MCS, mean (SD)^i^	52.4 (8.0)	53.7 (7.3)	51.8 (8.6)	52.3 (7.6)	.106

Bold values indicates statistically significant differences between the groups (*p* ≤ 0.05)

Continuous variables analyzed with ANOVA with Tukey’s HSD post hoc test or Kruskal–Wallis and Mann–Whitney U tests between pairs of groups

Categorical variables analyzed with x^2^-test. Significant differences between the marked groups: ^1^No pain versus Pain < 3 months, ^2^No pain versus Persistent pain and ^3^Pain < 3 months versus Persistent pain. *p* values marked with bold indicate statistically significant differences between the groups (*p* ≤ 0.05)

The pain group variable was recoded into three categories: “No pain,” “Pain < 3 months” (only once, < 1 month, 1–3 months) or “Persistent pain” (> 3 months, > 6 months, > 12 months)

*SD* standard deviation, *PCS* physical component summary, *MCS* mental component summary

^a^KIDSCREEN subscales

^b^Rasch scores were computed for each subscale and transformed into t-values with a mean of 50 and an SD of 10. Higher values indicate higher levels of HRQOL

^c^Range 1–4, where higher values indicate higher levels of self-efficacy

^d^Range 1–4, where higher values indicate higher levels of self-esteem

^e^Range 8–32, where higher values indicate higher levels of loneliness

^f^Range 0–1, where higher values indicate higher levels of stress

^g^The variable was dichotomized as “Usually/always” (usually, always) or “Sometimes/rarely” (sometimes, rarely, never)

^h^The variable was dichotomized as “No” or “Yes” (a slight problem, more than a slight problem, a big problem, a very big problem)

^i^RAND-36 scores range from 0 to range 0–100, where 100 means perfect health

The first part of the survey contained *sociodemographic information*. Adolescents answered questions about gender, age, adult members of the household, parents’ birthplace, parental work status and school absence. Parents answered questions about age, gender, marital status, education level, work status and household income. We used the variables regarding adult members of the household, parental education level and household income to indicate SES in further analyses.

*Pain in adolescents and in parents* was assessed using the Brief Pain Inventory (BPI), which asks participants to rate the subjective intensity of pain at its worst, least and in average, and how pain interferes with different aspects of life [47, 48]. Adolescents and parents answered questions about their own pain experiences. The items are presented as numeric rating scales, with 0 = no pain to 10 = pain as bad as you can imagine. For analysis, the interference items were combined into two indexes of interference: activity and emotions [47]. The Norwegian BPI has satisfactory psychometric properties and has been used among both adolescents and adults [48, 49]. We also used selected questions from the Lübeck Pain-Screening Questionnaire (LPQ), which assesses the presence and consequences of pain during the preceding 3 months [50]. Adolescents and parents were asked about pain duration and pain frequency. To be able to assess how selected factors were associated with adolescent pain groups, we used the adolescents’ pain duration variable to divide adolescents into three different groups. The variable was recoded into: “no pain,” “pain < 3 months” (only once, < 1 month or 1–3 months) or “persistent pain” (> 3 months, > 6 months or > 12 months). Additionally, the adolescents were asked about self-perceived triggers of pain. A list of possible causes (presented in the footnotes of Table 3) was derived from the LPQ questionnaire [50] and the adolescents were asked to tick all possible causes. The Norwegian LPQ has demonstrated satisfactory content validity and high internal consistency [8]. Only those who rated ≥ 1 on the “pain on average” question from the BPI (indicating that they had pain) were administered questions about pain interference (from the BPI) and questions about pain duration, frequency and self-perceived triggers of pain (from the LPQ). Finally, we used two questions derived from the Norwegian “Pain, Youth and Self-Medication study” [51, 52] to measure the intake of over-the-counter (OTC) analgesics in adolescents and in parents. First, the respondents were asked about OTC analgesic intake during the last 4 weeks. If they answered “yes,” they were asked about the frequency of intake.

**Table 3:** Descriptive pain characteristics of adolescents and parents by adolescent pain group (N = 508)

	Total (N = 508)	No pain (n = 124)	Pain < 3 months (n = 236)	Persistent pain (n = 148)	*p* value
*Adolescent characteristics*					
Pain worst, median (min, max)^a^	3.0 (0.0, 10.0)	0.0 (0.0, 10.0)	3.0 (0.0,9.0)	5.0 (0.0, 10.0)	**< .001**^**1,2,3**^
Pain least, median (min, max)^a^	0.0 (0.0, 8.0)	0.0 (0.0, 5.0)	1.0 (0.0, 8.0)	1.0 (0.0, 6.0)	**< .001**^**1,2**^
Pain average, median (min, max)^a^	2.0 (0.0, 10.0)	0.0 (0.0, 3.0)	2 (1.0, 10.0)	3 (1.0, 9.0)	**< .001**^**1,2,3**^
Pain interference on activity, median (min, max)^b,c^	1.3 (0.0, 10.0)		1.0 (0.0, 10.0)	1.7 (0.0, 9.0)	**< .001**^**3**^
Pain interference on emotions, median (min, max)^b,c^	1.2 (0.0, 9.2)		1.0 (0.0, 9.0)	1.4 (0.0, 9.2)	**.002**^**3**^
Pain frequency, N (%)^b,d^					**< .001**^**3**^
Seldom	159 (41.4)		148 (62.7)	11 (7.4)	
Sometimes	90 (23.4)		49 (20.8)	41 (27.7)	
Often	135 (35.2)		39 (16.5)	96 (64.9)	
Self-perceived triggers of pain^b,e^					
Emotions	78 (20.3)		44 (18.6)	34 (23.0)	.305
School	97 (25.3)		52 (22.0)	45 (30.4)	.066
Lack of sleep	99 (25.8)		59 (25.0)	40 (27.0)	.659
Cold/illness	58 (15.1)		45 (19.1)	13 (8.8)	**.006**^**3**^
Digital technology use	50 (13.0)		28 (11.9)	22 (14.9)	.395
Loneliness	115 (29.9)		58 (24.6)	57 (38.5)	**.004**^**3**^
Sport/physical activities	33 (8.6)		18 (7.6)	15 (10.1)	.393
Menstruation^f^	85 (35.6)		51 (36.2)	34 (34.7)	.815
Other	200 (52.1)		115 (48.7)	85 (57.4)	.097
Family members having pain, N (%)					**< .001**^**2,3**^
Yes	154 (30.4)	26 (21.1)	63 (26.7)	65 (43.9)	
Do not know	198 (39.1)	44 (35.8)	100 (42.4)	54 (36.5)	
No	155 (30.6)	53 (43.1)	73 (30.9)	29 (19.6)	
OTC analgesic intake during the last 4 weeks, N (%)					**.001**^**1,2**^
Yes	242 (47.7)	41 (33.3)	118 (50.0)	83 (56.1)	
No	265 (52.3)	82 (66.7)	118 (50.0)	65 (43.9)	
Frequency of OTC analgesic intake, N (%)^g,h^					.674
Every week	48 (19.8)	7 (17.1)	22 (18.6)	19 (22.9)	
Less often than every week	194 (80.2)	34 (82.9)	96 (81.4)	64 (77.1)	
*Parent characteristics*					
Pain worst, median (min, max)^a^	2.0 (0.0, 10.0)	1.0 (0.0, 10.0)	2.0 (0.0, 10.0)	2.0 (0.0, 9.0)	.138
Pain least, median (min, max)^a^	0.0 (0.0, 9.0)	0.0 (0.0, 5.0)	0.0 (0.0, 9.0)	0.0 (0.0, 9.0)	.529
Pain on average, median (min, max)^a^	1.0 (0.0, 10.0)	1.0 (0.0, 9.0)	1.0 (0.0, 9.0)	1.0 (0.0, 6.0)	.692
Pain interference on activity, median (min, max)^c,i^	0.7 (0.0, 10.0)	0.3 (0.0, 9.0)	0.7 (0.0, 10.0)	0.7 (0.0, 8.0)	.518
Pain interference on emotions, median (min, max)^c,i^	1.0 (0.0, 10.0)	0.5 (0.0, 9.0)	1.0 (0.0, 10.0)	1.0 (0.0, 8.0)	.465
Pain frequency, N (%)^d,i^					.944
Seldom	87 (28.2)	20 (27.4)	40 (27.6)	27 (30.0)	
Sometimes	58 (18.8)	13 (17.8)	30 (20.7)	15 (16.7)	
Often	163 (52.9)	40 (54.8)	75 (51.7)	48 (53.3)	
Pain duration, N (%)^i,j^					.963
No pain	200 (39.4)	51 (41.1)	91 (38.6)	58 (39.2)	
Pain < 3 months	100 (19.7)	24 (19.4)	49 (20.8)	27 (18.2)	
Persistent pain	208 (40.9)	49 (9.6)	96 (40.7)	63 (42.6)	
OTC analgesic intake during the last 4 weeks, N (%)					.661
Yes	296 (58.3)	68 (54.8)	141 (59.7)	87 (58.8)	
No	212 (41.7)	56 (45.2)	95 (49.3)	61 (41.2)	
Frequency of OTC analgesic intake, N (%)^h,k^					.587
Every week	96 (32.4)	21 (30.9)	43 (30.5)	32 (36.8)	
Less often than every week	200 (67.6)	47 (69.1)	98 (69.5)	55 (63.2)	

Bold values indicates statistically significant differences between the groups (*p* ≤ 0.05)

Continuous variables analyzed with Mann–Whitney U test or Kruskal–Wallis with Mann–Whitney U tests between pairs of groups

Categorical variables analyzed with x^2^-test. Significant differences between the marked groups: ^1^No pain versus Pain < 3 months, ^2^No pain versus Persistent pain and ^3^Pain < 3 months versus Persistent pain. *p* values marked with bold indicate statistically significant differences between the groups (*p* ≤ 0.05)

The pain group variable was recoded into three categories: “No pain,” “Pain < 3 months” (only once, < 1 month, 1–3 months) or “Persistent pain” (> 3 months, > 6 months, > 12 months)

*OTC* over the counter

^a^Range 0–10, where 10 indicates pain as bad as you can imagine

^b^N = 384

^c^Range 0–10, where 10 indicates complete interference of pain

^d^The variable was recoded into three categories: “seldom” (< once/month, once/month), “sometimes” (2–3 times/month, once/week) or “often” (several times/week, every day)

^e^The variable was recoded into nine categories: “Emotions” (anger/disputes, sadness, agitation), “School” (school situation, school work), “Lack of sleep,” “Cold/illness,” “Digital technology use” (social media, screen time), “Loneliness,” “Sport/physical activities,” “Menstruation” and “Other” (change of weather, noise, family condition, a new situation, nutrition/sweets, nonspecific factors)

^f^N = 239 (only girls were asked about this possible trigger of pain)

^g^N = 242

^h^The variable was dichotomized as “Every week” (daily, every week but not daily) or “Less often than every week” (less often than every week, no intake)

^i^N = 308

^j^The variable was recoded into three categories: “No pain,” “Pain < 3 months” (only once, < 1 month, 1–3 months) or “Persistent pain” (> 3 months, > 6 months, > 12 months)

^k^N = 296

*HRQOL in adolescents* was assessed using the KIDSCREEN-27 questionnaire [53, 54]. The KIDSCREEN-27 is a multidimensional measure of generic HRQOL and consists of 27 questions grouped into five subscales: (1) physical well-being, (2) psychological well-being, (3) autonomy and parent relations, (4) social support and peers and (5) school environment [53, 55, 56]. The KIDSCREEN questionnaire is scored on a 1–5 Likert scale referring to the last week. The scale indicates either the intensity of an attitude or the frequency of certain behaviors or feelings. In line with the KIDSCREEN-handbook [55], Rasch scores were computed for each subscale and transformed into t-values that can be compared with international t-values. These t-values are normed to a mean (SD) of 50 (10) [55]. The answers were recoded so that higher scores indicate better HRQOL. The Norwegian version of the KIDSCREEN-27 has been shown to be reliable and valid [54].

*HRQOL in parents* was assessed using the 36-item Medical Outcomes Study Short Form (RAND-36), which consists of eight domains (general health, bodily pain, physical function, role limitations [physical], mental health, vitality, social function and role limitations [emotional]). These eight domains can be combined into a physical component summary scale (PCS) and a mental component summary scale (MCS), which reflect physical and mental health, respectively [57, 58]. We used the PCS and MCS scales in this study. The RAND-36 scales were scored according to recommended scoring procedures, and sum scales were expressed using values from 0 to 100, with 100 representing excellent health [57, 58].

*Self-efficacy in adolescents* was assessed using the 10-item Generalized Self‐Efficacy scale (GSE), which measures optimistic self-beliefs in coping with the tasks, demands and challenges of life in general [59, 60]. The scale includes 10 statements that the respondent rates on a 1–4-point scale. The respondent’s scores on each item are summed and divided by 10 to obtain a GSE score ranging from 1 to 4. Higher scores indicate higher levels of GSE. The GSE has been found to be valid and reliable, with satisfactory internal consistency [40, 59].

*Self-esteem in adolescents* was assessed using the four-item version of the Rosenberg Self-Esteem Scale (RSES) [61, 62] consisting of four statements on self-perceptions related to attitude toward oneself, feeling of uselessness, having something to be proud of, and self-worth. RSES is rated on a 1–4 Likert scale. Scores are summed and divided by 4 to obtain a RSES score ranging from 1 to 4, where higher scores represent higher self-esteem. The four-item version correlates highly with the original 10-item version (0.95) [62], which is considered a valid measure of self-esteem in a large body of literature. The four-item version has previously been used among Norwegian adolescents and has good internal consistency [63–65].

*Loneliness in adolescents* was assessed using the eight-item version of the revised UCLA Loneliness Scale (ULS-8), which is considered to be a reliable and adequate measure of loneliness among adolescents [66–68]. The eight items are rated on a 1–4-point scale, with values ranging from “never” to “always.” The total score ranges from 8 to 32 points, with higher scores indicating higher levels of loneliness. The Norwegian version of ULS-8 has been found reliable with satisfactory internal consistency [43].

*Stress in adolescents* was assessed using the 30-item Perceived Stress Questionnaire (PSQ) [69–71]. PSQ refers to the last 4 weeks and is rated on a 4-point rating scale. PSQ contains both negatively and positively formulated items to reduce acquiescent bias. The answers were recoded so that higher scores indicate higher levels of perceived stress. The resulting PSQ total score is transformed linearly between 0 and 1: PSQ = (raw value − 30)/90. Commonly applied cutoff levels of stress within PSQ are low: < 0.33, medium: 0.33–0.45, moderate: 0.45–0.60 and severe: > 0.60 [69]. The Norwegian version of PSQ has shown good reliability and validity [71].

*Sleep* was assessed using two questions derived from the School Sleep Habits Survey, which has been widely used in adolescents [72]. We used one question focusing on frequency of enough sleep and one focusing on problems with sleepiness during daily activities. The School Sleep Habits Survey has an established validity compared to sleep diaries and actigraphy [73] and has previously been used among Norwegian adolescents [74].

### Data analyses

The statistical analyses were conducted using IBM SPSS Statistics (version 26.0). Descriptive statistics were calculated for all variables according to three previously defined adolescent pain groups. Continuous variables were described with mean and standard deviation or as median and min/max, categorical variables with counts and percentages. Strength of associations between pairs of selected variables was assessed using chi-square test for categorical data and ANOVA with Tukey’s HSD post hoc test or Kruskal–Wallis and Mann–Whitney U tests for continuous data. To fulfill the assumption of “minimum expected cell frequency” for chi-square analyses [75], some of the items that had several categories were recoded into fewer categories.

**Fig. 1:**
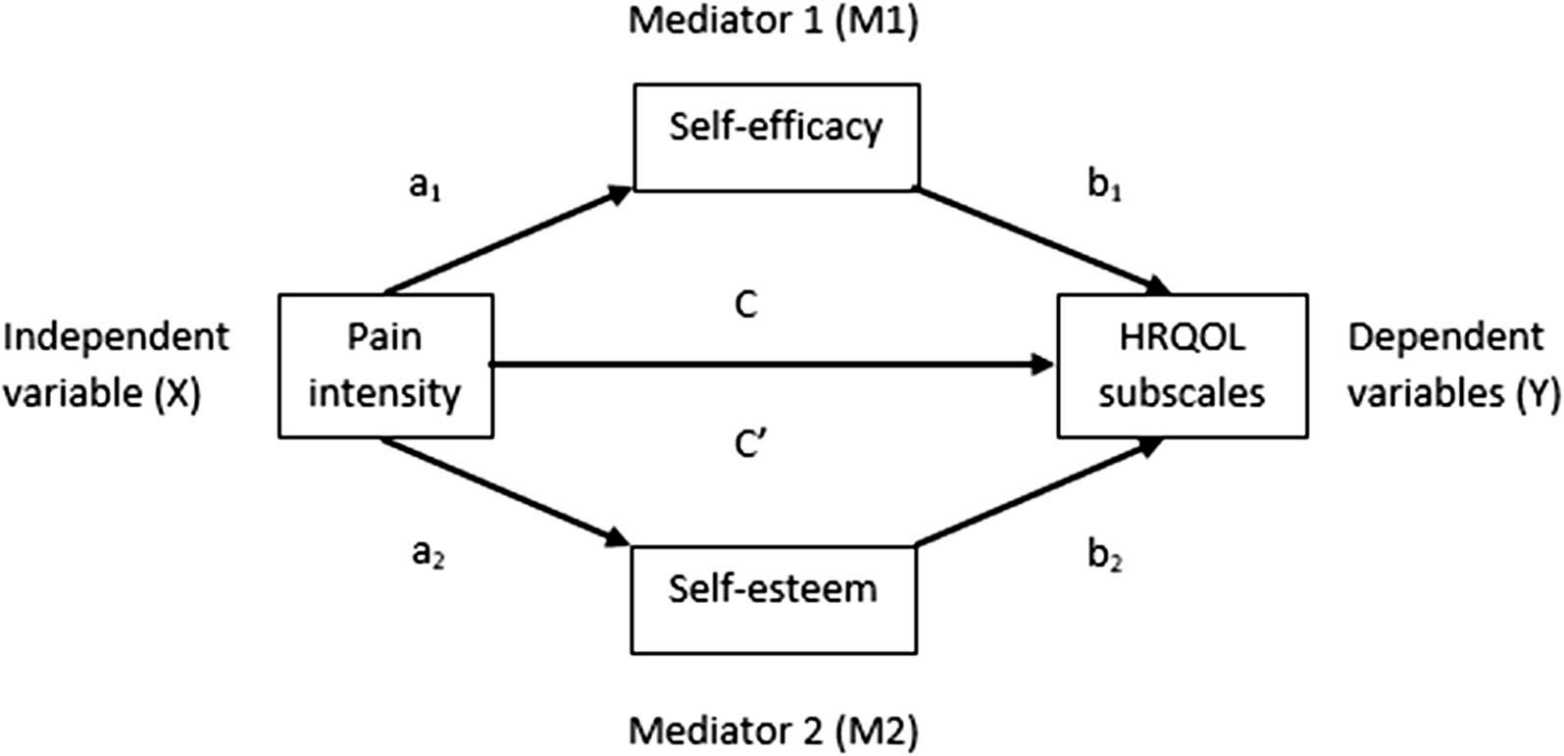
Schematic of our parallel multiple mediation model

Mediation analyses were conducted using the PROCESS macro method developed for SPSS by Hayes [76]. We proceeded using the parallel multiple mediation model depicted in Fig. 1. PROCESS was used to estimate (1) the direct effect of pain on HRQOL (C′), (2) the total effect of pain on HRQOL (C) and (3) the specific indirect effects through mediator 1 (self-efficacy) and mediator 2 (self-esteem) (a_i_b_i_). Gender, adult members of the household, parental education and household income were entered as covariates. Proportions mediated for the direct and indirect effects were estimated as the direct effect/total effect and the indirect effect/total effect and multiplied by 100 to be interpreted as percentages. The calculation of direct and total indirect effect as percentages was not applicable for the HRQOL subscales autonomy and parent relations and social support and peers due to opposite directions of the total effects and the direct effects. The mediation effect was considered statistically significant if the 95% confidence interval (CI) for the effect did not include zero. All the analyses were considered exploratory. Hence, no correction for multiple testing was done, and *p* values < 0.05 were considered significant. All the tests were two-sided. Assumptions for mediation analyses were checked and fulfilled. According to Preacher and Hayes, a significant indirect effect is no longer seen as a prerequisite for mediation [77]. Thus, all HRQOL subscales were included.

## Results

### Descriptive data for sociodemographic variables in adolescents and their parents

In total, 508 dyads of adolescents with one parent each participated in the study. The majority were girls (55.3%) and mothers (77.4%), respectively. The adolescents’ ages ranged from 14 to 15 years, with a median age of 14 years. The mean (SD) age for the parents were 45.2 (4.9) years. Among the adolescents, 148 had persistent pain. The three adolescent pain groups were similar concerning all the selected sociodemographic variables in adolescents and their parents, except for adolescents’ gender and school absence (Table 1).

### Descriptive data for adolescent- and parent pain-related factors

Descriptive data for adolescent- and parent pain-related factors by adolescent pain groups are presented in Table 2. Adolescents with pain lasting less than 3 months and persistent pain reported significantly higher levels of stress, loneliness and lack of sleep and lower levels of self-efficacy, self-esteem and HRQOL compared to adolescents without pain. Adolescents with persistent pain reported significantly lower HRQOL than adolescents with pain lasting less than 3 months for the KIDSCREEN subscales physical well-being, psychological well-being and autonomy and parent relations. Moreover, adolescents with persistent pain reported significantly lower levels of self-esteem and significantly higher levels of loneliness, stress and lack of sleep than adolescents with pain lasting less than 3 months. Considering the parents’ HRQOL, there were no statistically significant differences between the adolescent pain groups.

### Descriptive pain characteristics of adolescents and parents

Table 3 shows the descriptive pain characteristics of adolescents and parents by adolescent pain groups (Table 3). Adolescents with persistent pain experienced pain significantly more often and reported significantly higher values of the worst pain and average pain compared to adolescents with pain lasting less than 3 months. Moreover, adolescents with persistent pain also reported significantly higher levels of pain interference in both activity and emotions.

The most prevalent self-perceived triggers of pain, as reported by the adolescents, were loneliness (29.9%), lack of sleep (25.8%) and school (25.3%). Girls also rated menstruation (35.6%) as a prevalent trigger of pain. More adolescents with persistent pain (38.5%) reported loneliness as a trigger compared to adolescents with pain lasting less than 3 months (24.6%).

Almost two-thirds of the adolescents and more than half of the parents with pain experienced pain in multiple body locations. Head pain and neck/shoulder pain were the most common pain locations for both adolescents and the parents. Among both adolescents and parents, about half reported intake of OTC analgesics during the last 4 weeks. Among these, almost one-third of the parents reported intake every week, while about 20% of the adolescents reported intake every week. Our univariate analyses showed no significant relationship between the adolescents’ and their parents’ use of OTC analgesics.

There were no statistically significant differences between the adolescent pain groups concerning any of the parents’ pain characteristics. However, more adolescents with persistent pain (43.9%) reported that someone in their family had pain compared to adolescents with pain lasting less than 3 months (26.7%) and adolescents without pain (21.1%).

### Mediation by self-efficacy and self-esteem on the relationship between pain intensity and HRQOL in adolescents with persistent pain

**Fig. 2:**
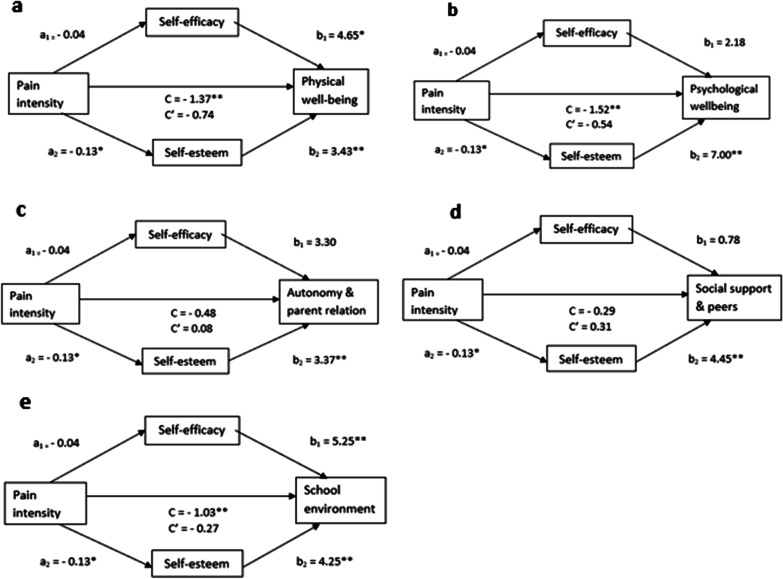
Parallel multiple mediation by self-efficacy and self-esteem of the association between pain and HRQOL subscales in adolescents with persistent pain. Gender, Adult members of the household, Parental education and Household income were entered as covariates. **a** Physical well-being, **b** Psychological well-being, **c** Autonomy and parent relation, **d** Social support and peers and **e** School environment. *HRQOL* health-related quality of life. *p < 0.05, **p < 0.01. Path a and b represents the indirect effects through the mediators. Path C′ depicts the direct effect and C the total effect

We have suggested a parallel multiple mediation by self-efficacy (M1) and self-esteem (M2) of the association between pain intensity (X) and the scores for HRQOL subscales (Y) in adolescents with persistent pain, as depicted in Fig. 2. Unstandardized estimates of the Bs of the associated variables are depicted in the figure. We found that pain was associated with decreased self-efficacy (a_1_ = − 0.04) and self-esteem (a_2_ = − 0.13). However, the associations were only significant for self-esteem. Further, we found that having a higher self-efficacy score (b_1_) was significantly associated with higher HRQOL scores for the subscales physical well-being (b_1_ = 4.65) and school environment (b_1_ = 5.25). Moreover, we found that having a higher self-esteem score (b_2_) was significantly associated with higher HRQOL scores for all the subscales: physical well-being (b_2_ = 3.43), psychological well-being (b_2_ = 7.00), autonomy and parent relations (b_2_ = 3.37), social support and peers (b_2_ = 4.45) and school environment (b_2_ = 4.25).

The total effect (C) of pain on HRQOL was statistically significant for the subscales physical well-being (C = − 1.37), psychological well-being (C = − 1.52) and school environment (C = − 1.03). This indicates that for these subscales, an increased pain score is associated with a decreased HRQOL score after adjusting for the two mediators and holding the covariates gender, adult members of the household, parental education and household income constant. The direct effect (C′) of pain on HRQOL was no longer statistically significant for any of the subscales, indicating that the association were completely mediated by self-efficacy and self-esteem.

Table 4 shows the reduction in HRQOL subscales (presented as percentages) in adolescents with persistent pain explained by the direct (pain intensity) and indirect (self-efficacy and self-esteem) effects. Self-efficacy was not a mediator in the relationship between pain and HRQOL. Self-esteem completely mediated the relationship between pain and HRQOL for the subscales physical well-being, psychological well-being and school environment. More than half of the reductions in the HRQOL subscale scores for psychological well-being and school environment, and about one-third of the reduction for physical well-being were explained by the mediating variable self-esteem. Among the five HRQOL subscales, the total indirect effect was highest for the subscale school environment (73.5%).

**Table 4:** Reduction in HRQOL subscales in adolescents with persistent pain explained by direct (pain intensity) and indirect (self-efficacy and self-esteem) effects

	Direct effect	Indirect effect of self-efficacy	Indirect effect of self-esteem	Total indirect effect^a^
Physical well-being^b^	54.2%	13.7%	32.1%*	45.8%*
Psychological well-being^b^	35.4%	5.7%	58.8%*	64.6%*
Autonomy and parent relation^b^	–	–	–	–
Social support and peers^b^	–	–	–	–
School environment^b^	26.5%	20.5%	53.0%*	73.5%*

*HRQOL* health-related quality of life

^*^Statistically significant; the 95% confidence interval for the effect did not include zero

^a^The specific indirect effect of self-efficacy + the specific indirect effect of self-esteem (a_1_b_1_ + a_2_b_2_)

^b^KIDSCREEN subscales

## Discussion

Overall, we believe our findings support the need to understand pain through a holistic model such as the multidimensional biobehavioral model of pediatric pain [44, 45]. In light of this model, self-esteem, self-efficacy, stress, loneliness, sleep and sociodemographic factors might serve as both precipitants that pain may arise from and intervening variables that can influence both pain and HRQOL. Specifically, we believe our results highlight that self-esteem and self-efficacy should be considered as important intervening variables.

### The complexity within adolescent pain

The current findings demonstrate the complexity within adolescent pain. We found that adolescents with pain differ from adolescents without pain when it comes to gender and school absence and factors within-person (self-efficacy, self-esteem, stress, sleeping problems) and between-persons (loneliness). Further, in line with previous studies [3, 38], this study shows that having pain is negatively associated with HRQOL, indicating that pain affects physical, psychological, social and functional aspects of adolescents’ lives. As highlighted in another Norwegian study [8], pain problems seem to have widespread and generally negative effects on several aspects of adolescents’ lives. We also found a wide variety considering the adolescents’ self-perceived triggers of pain, emphasizing the subjectivity within pain experiences.

Our results demonstrate that adolescents with persistent pain constitute a vulnerable group, as they reported higher levels of stress, loneliness, lack of sleep and lower levels of self-efficacy, self-esteem and HRQOL compared to adolescents with less pain. Negative findings related to having persistent pain have also been highlighted in other studies [4, 7, 22]. Further, our results indicate that a longer pain duration is making the adolescents more vulnerable to pain interference.

It is notable that in addition to reporting higher scores of loneliness, more adolescents with persistent pain reported loneliness as a self-perceived trigger of pain compared to adolescents with a shorter pain duration. This indicate that loneliness is a significant problem among adolescents with persistent pain. Our findings are in line with the review of Forgeron et al. [9], who found that adolescents with persistent pain have peer relationship deficiencies. Across studies, this review found that adolescents with persistent pain were viewed as more isolated than healthy peers, were reported to have fewer friends and may be subjected to more peer victimization. Loneliness is strongly associated with HRQOL in adolescents [43]. Thus, an increased awareness of peer relationship challenges for adolescents with persistent pain is very important.

### The mediating role of self-esteem and self-efficacy on the relationship between pain and HRQOL in adolescents with persistent pain

The effect of pain on HRQOL was completely mediated by self-esteem but not by self-efficacy, indicating that self-esteem plays a more important role than self-efficacy on the relationship between pain and HRQOL. Our findings contrast with the study of Grasaas et al. [38], who found that the associations between pain intensity and several HRQOL subscales were mediated by self-efficacy in adolescents with persistent pain. However, unlike Grasaas et al.’s study, our study included self-esteem as a parallel mediator and controlled for gender as a possible confounder, which may explain the different results. Previous studies have found important gender differences among adolescents when it comes to pain, HRQOL, self-esteem and self-efficacy [13, 35, 38, 43]. This may explain our results.

The review of Cousins and colleagues ([18] pp. 843–844) highlights that “the concept of resilience empowers youth to foster their skills and strengths to positively adapt and live successfully with their pain,” and previous research have highlighted the importance of considering high self-esteem and self-efficacy as important protective or resource factors for HRQOL in adolescents [35–39, 43]. Our results confirm the importance of resilience factors for well-being in adolescents with persistent pain by showing that up to 73.5% of the reduction in the HRQOL subscale scores for physical well-being, psychological well-being and school environment could be explained by the mediating variables self-esteem and self-efficacy. Furthermore, the total indirect effect was highest for the HRQOL subscale school environment, indicating that self-esteem and self-efficacy especially play an important part in adolescents with pain’s well-being at school. Previous studies have found that low self-esteem relate to relative increases in loneliness over time and vice versa [78]. Peer relationship is important at school and adolescents experience relationships with peers as vital to their well-being [79]. Considering that loneliness was found to be a significant problem among the adolescents with persistent pain, this might explain our findings. Another noteworthy finding was that the associations between the HRQOL subscales and self-esteem were statistically significant for all five HRQOL subscales, while self-efficacy was only significantly associated with the subscales physical well-being and school environment. However, for these two subscales, self-efficacy had a higher effect than self-esteem. This shows that self-esteem and self-efficacy affect physical, psychological, social and functional aspects of adolescents’ lives in different ways, suggesting that both resilience factors are important for the well-being of adolescents.

Resilience factors such as self-esteem and self-efficacy are especially important during adolescence. The social and emotional development of adolescents is characterized by a struggle with sense of identity, where adolescents strive for independence from caregivers while becoming increasingly influenced by peers [80]. Support from family and friends is considered vital to enhance resilience [33]. To increase the sense of self-esteem and self-efficacy, Stewart and Yuen [33] recommends that individuals should be encouraged to think of other challenging situations they have mastered. According to their review, encouraging a sense of hope, realistic optimism and mastery over either the problem (e.g. pain) or one’s ability to cope with it might be helpful.

### Family influence on adolescent pain

Parent history of pain may increase adolescent risk for chronic pain [14, 15, 23]. Surprisingly, our study found no differences between the adolescent pain groups considering any of the parents’ pain characteristics. However, more adolescents with persistent pain reported that someone in their family had pain, indicating that family history of pain still plays a significant role. It is possible that other elements in the family environment may have contributed to our results considering that the relationship between parental and adolescent pain may be a result of complex interactions between genetics, environmental factors and learned pain behavior [14–17]. Parent factors may shape adolescents’ pain development and pain management from both a resilience perspective and a risk perspective [16, 18, 19], and differences in adolescents’ attachment to their caregivers and how they communicate about pain might affect how adolescents experience and manage pain [81].

We found that about half of adolescents and parents reported intake of OTC analgesics and that parents reported more frequent intake compared to the adolescents. Considering the relatively low intensity of pain reported, this might indicate that adolescents and parents use OTC analgesics for reasons other than only pain relief. According to Skarstein et al.’s [82] review, parents are the most important source of information regarding adolescents’ use of OTC analgesics, as well as the main supplier. Thus, our findings highlight that informing parents, adolescents, and society about how to use OTC analgesics appropriately should be a high priority.

Contrary to previous studies that have shown that children from families with low SES experience pain more frequently [2, 20, 21], we found that the three adolescent pain groups were similar concerning SES factors such as adult members of the household, parental work status, parental education level and household income. A possible explanation for this might be that our sample mainly consisted of adolescents from families with higher levels of SES. However, it is important to highlight that although significant shared effects between family members (e.g., economy, education, cohabitant status) is associated with chronic pain, most explanations for chronic pain are considered to be at the individual level [83].

### Strengths and limitations

The main strengths of this study include the relatively large sample of adolescent–parent dyads recruited from a variety of schools and that the selected analyzed variables were assessed with well-validated instruments. The limitations of this study include the cross-sectional design, which makes causal inference challenging to determine. Further, our meditation analyses are of an exploratory nature and based on our assumptions and understanding of the current research area. Thus, we can assume the direction of the indirect and direct effects. Another limitation is linked to the low response rate. We do not have information about the nonparticipating adolescents and parents. Therefore, we cannot assess whether the nonparticipants and participants differed in any way. Also, we only included one of each adolescent’s parents, which may have affected the results. Hence, the inclusion of both parents is recommended in future studies. However, more than three quarters of the adolescents lived with both parents and had parents who were both working. Further, among the participating parents, around three quarters had higher education level, were working full time and had a household income of more than 750,000 NOK/year. This indicates that the results may not be representative of adolescents within families with lower SES, which should be considered when interpreting our results. Further, the pain intensity reported by the adolescents is not considered high, which indicates that the results may not be representative of adolescents with higher levels of pain. Moreover, sample size and heterogeneity in the sample may have led to self-efficacy not being significant in our mediation analyses. We can therefore not exclude a possible significant influence from self-efficacy even if we were not able to show it in our study. Thus, we recommend a larger sample in future studies.

### Implications

This study contributes to more knowledge of factors that characterizes adolescents with and without pain in a school-based setting, which can help teachers, public health nurses, parents and researchers better understand the cause of pain problems and find the best strategies to help adolescents with pain. It seems nearly impossible to focus solely on singular factors when helping adolescents to cope with pain. Thus, we recommend an individual, holistic approach to adolescents’ pain. In order to have an increased focus on protective and resilience factors, we suggest that public health nurses include routine-questions about self-esteem and self-efficacy in their consultations with adolescents who experience pain. Moreover, considering that the current COVID-19 pandemic is associated with increased stress and loneliness in adolescents [84, 85] parents, teachers, health professionals and researchers should be aware of the risk for an increase in pain problems among adolescents during and after the pandemic.

Based on our results from the mediation analyses, we recommend that HRQOL-promoting interventions among adolescents with pain should focus on a strengthening of their self-esteem and self-efficacy. It is demanding to intervene on risk-factors associated with adolescents’ pain such as SES, stress, and parental factors. However, interventions aimed at increasing self-esteem and self-efficacy is promising and also possible to carry out, for instance in a school setting, and should thus be a high priority. Considering that the basis for self-esteem and self-efficacy is founded during childhood, we recommend that a strengthening of these resilience factors should be a focus in early age, not only when reaching adolescence. We suggest the school setting as an important arena for resilience-promoting interventions.

## Conclusions

This cross-sectional study among 14–15-year-old adolescents demonstrates the complexity and subjectivity within adolescent pain and shows that adolescents with pain differ from adolescents without pain when it comes to gender and school absence and factors within-person (self-efficacy, self-esteem, stress, sleeping problems) and between-persons (loneliness). We found no statistically significant differences between the adolescent pain groups, considering the selected parental factors; however, more adolescents with persistent pain reported that someone in their family had pain. Our results emphasize that longer pain duration makes adolescents more vulnerable, especially considering peer relationship. Furthermore, the results of our mediation analyses confirm the importance of resilience factors for HRQOL in adolescents with persistent pain but indicate that self-esteem plays a more important role than self-efficacy. Still, to promote HRQOL in adolescents with persistent pain, we suggest a strengthening of both their self-esteem and self-efficacy. We highlight the need for an individual, holistic approach to adolescent pain.

## Supplementary Information

**Additional file 1.** Cronbach’s alpha values for instruments used in this study.

## Data Availability

The datasets used and/or analyzed during the current study are not publicly available due to General Data Protection Regulation laws but are available from the corresponding author on reasonable request and with permission from the Norwegian Centre for Research Data.

## Abbreviations

BPI: Brief pain inventory
GSE: General self-efficacy scale
HRQOL: Health-related quality of life
LPQ: Lübeck pain-screening questionnaire
MCS: Mental component summary scale
OTC: Over the counter
PCS: Physical component summary scale
PSQ: Perceived stress questionnaire
RSES: Rosenberg self-esteem scale
SES: Socioeconomic status
RAND-36: The 36-item medical outcomes study short form
ULS: UCLA loneliness scale
