# Supplementary material for: Pain and health-related quality of life in adolescents and the mediating role of self-esteem and self-efficacy: a cross-sectional study including adolescents and parents
Source: BMC Psychol. 2021 Aug 30;9:128. doi: 10.1186/s40359-021-00629-z (PMC8404017; doi:10.1186/s40359-021-00629-z)
Supplement: Supplementary file 1 — Additional file 1. Cronbach’s alpha values for instruments used in this study. [file 40359_2021_629_MOESM1_ESM.docx]

**Additional file 1: Cronbach’s alpha values for instruments used in this study**

| Factors | Instruments | Number  of items | α ^ab^ | α ^ac^ |
| --- | --- | --- | --- | --- |
| Pain | Brief Pain Inventory |  |  |  |
|  | Pain interference on activity | 3 | 0.77 | 0.88 |
|  | Pain interference on emotions | 4 | 0.89 | 0.90 |
| HRQOL | KIDSCREEN-27 |  |  |  |
|  | Physical well-being | 5 | 0.81 |  |
|  | Psychological well-being | 7 | 0.88 |  |
|  | Autonomy and parent relations | 7 | 0.77 |  |
|  | Social support and peers | 4 | 0.79 |  |
|  | School environment | 4 | 0.80 |  |
|  | RAND-36 |  |  |  |
|  | Mental health |  |  | 0.81 |
|  | Vitality |  |  | 0.89 |
|  | Bodily pain |  |  | 0.85 |
|  | General health |  |  | 0.83 |
|  | Social function |  |  | 0.87 |
|  | Physical function |  |  | 0.90 |
|  | Role limitation (physical) |  |  | 0.93 |
|  | Role limitation (emotional) |  |  | 0.88 |
| Self-efficacy | Generalized Self‐Efficacy Scale | 10 | 0.88 |  |
| Self-esteem | Rosenberg Self-Esteem Scale | 4 | 0.81 |  |
| Loneliness | UCLA Loneliness Scale | 8 | 0.80 |  |
| Stress | Perceived Stress Questionnaire | 30 | 0.93 |  |

ª Cronbach’s alpha coefficient values in this study, ^b^ in adolescents, ^c^ in parents

HRQOL, health-related quality of life
